# Increased Oral Detection, but Decreased Intestinal Signaling for Fats in Mice Lacking Gut Microbiota

**DOI:** 10.1371/journal.pone.0039748

**Published:** 2012-06-29

**Authors:** Frank A. Duca, Timothy D. Swartz, Yassine Sakar, Mihai Covasa

**Affiliations:** 1 Institut National de la Recherche Agronomique, Centre de Recherche de Jouy-en-Josas, UMR 1319, MICALIS, Neurobiology of Ingestive Behavior, Domaine de Vilvert, Jouy-en-Josas, France; 2 University Pierre and Marie Curie, Paris, France; 3 Western University of the Health Sciences, College of Osteopathic Medicine, Basic Medical Sciences Department, Pomona, California, United States of America; National Institute of Agronomic Research, France

## Abstract

Germ-free (GF) mice lacking intestinal microbiota are significantly leaner than normal (NORM) control mice despite consuming more calories. The contribution of microbiota on the recognition and intake of fats is not known. Thus, we investigated the preference for, and acceptance of, fat emulsions in GF and NORM mice, and associated changes in lingual and intestinal fatty acid receptors, intestinal peptide content, and plasma levels of gut peptides. GF and NORM C57Bl/6J mice were given 48-h two-bottle access to water and increasing concentrations of intralipid emulsions. Gene expression of the lingual fatty acid translocase CD36 and protein expression of intestinal satiety peptides and fatty-acid receptors from isolated intestinal epithelial cells were determined. Differences in intestinal enteroendocrine cells along the length of the GI tract were quantified. Circulating plasma satiety peptides reflecting adiposity and biochemical parameters of fat metabolism were also examined. GF mice had an increased preference and intake of intralipid relative to NORM mice. This was associated with increased lingual CD36 (P<0.05) and decreased intestinal expression of fatty acid receptors GPR40 (P<0.0001), GPR41 (P<0.0001), GPR43 (P<0.05), and GPR120 (P<0.0001) and satiety peptides CCK (P<0.0001), PYY (P<0.001), and GLP-1 (P<0.001). GF mice had fewer enteroendocrine cells in the ileum (P<0.05), and more in the colon (P<0.05), relative to NORM controls. Finally, GF mice had lower levels of circulating leptin and ghrelin (P<0.001), and altered plasma lipid metabolic markers indicative of energy deficits. Increased preference and caloric intake from fats in GF mice are associated with increased oral receptors for fats coupled with broad and marked decreases in expression of intestinal satiety peptides and fatty-acid receptors.

## Introduction

By the year 2030, half of the American adult population is predicted to be obese, which is attributed primarily to increased caloric intake [1]. As such, the large contribution of calories from dietary fats may play a major role in the development of obesity. Despite the strong link between dietary fat intake and obesity, the factors leading to the over consumption of, and preference for, fats are less clear, but may be due to oral, intestinal, and metabolic influences. For example, rats rapidly consume oils during sham feeding, a process that limits post-oral feedback [2], while post-oral infusion of fat conditions flavor preferences in rats and mice [3], [4]. Furthermore, animals efficient in fat digestion or metabolism consume more fat than inefficient fat digesting and metabolizing counterparts [5]. Intestinal and metabolic factors are profoundly influenced and modulated by the presence of trillions of microbes residing in the intestinal tract, collectively referred to as the gut microbiota, which contribute to altered energy intake and increased adiposity. Recent studies have linked the gut microbiota to obesity and associated alterations in metabolism. For example, germ-free (GF) animals, lacking gut microbiota, are significantly leaner on a standard rodent chow diet than normal (NORM) animals with an intact microbiota despite consuming more energy [6]. Furthermore, most studies show that GF mice are resistant to diet-induced obesity from a high-fat (HF)- or western diet [7], [8], although in one recent study; albeit in a different strain, GF mice gained more weight and body fat than NORM mice on a calorically similar HF-diet but differing ingredient composition [9]. The resistance to fat deposition in GF mice appears to be due to several mechanisms, including decreased hepatic *de novo* lipogenesis. As well, increased systemic lipolysis through increased expression of fasting induced adipocyte factor (FIAF), an intestinal lipoprotein lipase (LPL) inhibitor which results predominantly from decreased extraction of energy from the diet [7], may play a role in the protection from obesity in GF mice, although the role of FIAF in the relationship between gut colonization and adiposity has been recently disputed (see [9]). In addition to influencing host metabolism, the absence of gut microbiota leads to alterations in intestinal morphology and physiology. We have recently demonstrated that GF mice exhibit increased “sweet” nutrient receptors and, sodium glucose-like transporter 1 (SGLT1) expression in the proximal intestine which was associated with increased sucrose intake [10]. The contribution of nutrient receptors to increased caloric intake in GF animals is not known, however, activation of nutrient responsive receptors leads to release of intestinal satiety peptides, such as cholecystokinin (CCK), glucagon-like peptide-1 (GLP-1), and peptide YY (PYY) [11]–[13]. Further evidence linking the gut microbiota to intestinal satiety peptides is the demonstration that GF mice conventionalized with donor microbiota display an increase in plasma PYY [11], while prebiotic treatment increases circulating GLP-1 and PYY with concomitant decreases in plasma ghrelin [14]. Together, these results suggest that alterations in nutrient sensing and peptide hormones influencing fat ingestion due to lack of microbiota may result in altered fat intake in GF animals.

In addition to the influence of intestinal nutrient sensing on long-term consumption of dietary fats, oral factors also play an important role in the detection of, and preference for, fats. As such, mice lacking CD36, a putative fatty-acid translocase located on the posterior lingual epithelium, are unable to develop preferences for low concentrations of oil [15]. Interestingly, expression of CD36 is determined by a variety of factors, including diet and energy status. For example, obese and non-obese animals consuming a HF-diet display decreased expression of CD36 compared to LF-fed or non-obese controls [16]. Conversely, during fasting, mice exhibit increased expression of CD36, an energy state associated with increased detection of fats [16], [17]. Because GF mice display marked reduction in adiposity, reflecting a state of energy deprivation, they may also display increased CD36, leading to increased detection or consumption of fats. Therefore, to examine the impact of the absence of the microbiota on fat intake and preference we first employed two-bottle 48-h access to increasing concentrations of intralipid emulsions in GF and NORM C57Bl/6J mice. Secondly, to assess changes in fat detection components and possible mechanisms involved in increased caloric intake, we measured expression of fatty acid sensors and receptors in the lingual and proximal intestine epithelium as well as peptide content and circulating satiety peptide levels in GF and NORM mice. Finally, we measured plasma lipid metabolites and quantified the enteroendocrine cells in the proximal (duodenum, jejunum) and distal (ileum, colon) intestine of both groups.

## Methods

### Animals

Throughout all experiments, male C57BL/6J GF mice (n = 10) from our germ-free colonies, originally derived from Charles River colonies (ANAXEM, Jouy-en-Josas, France), and normal (NORM) mice (n = 10) (Charles River, France) were housed individually in polycarbonate cages with cedar bedding. Each group (GF or NORM) was housed separately in two Trexler-type isolators (Igenia, France). Throughout the studies, sterility of the germ-free isolator was verified through weekly analysis of mouse fecal samples. Both groups of mice received similar autoclaved, deionized water and irradiated standard rodent chow (Safe Diets, Belgium) *ad libitum,* unless noted otherwise. They were allowed a minimum of one-week acclimation before experimental manipulations began. Procedures were carried out in accordance with the European Guidelines for the Care and Use of Laboratory Animals.

### 48-h Two-bottle Preference Tests

Ten-wk old GF and 9-wk old NORM mice weighing 27.1±0.3 g and 24.5±0.5 g respectively, were given access to intralipid emulsions that were prepared on the basis of percentage of soybean oil (0.156, 0.313, 0.626, 1.25% oil (v/v)) in ascending concentrations, and water during 48-h two-bottle testing. Due to technical logistics in GF isolators, only eight out of the 10 rats for each group were used for behavioral testing. At the beginning of each test, mice were weighed, and the water was removed and replaced with 2 similar 250-ml plastic bottles with the spouts penetrating from the top floor of the cage at 2–4 cm distance from the floor and 5–6 cm apart. The positions of the two bottles were alternated every 24-h to control for side preference. Bottles were weighed at the beginning and end of each 24-h test. Between each test, mice received one-bottle access to water. In previous experiments using the same bottles, we found that spillage from water bottles was negligible, therefore we did not account for spillage. Emulsions were presented once every 3–5 days, giving mice access to emulsions at least once a week. At no time did the mice receive more than two intralipid emulsion concentrations per week. To account for the fact that mice may have altered caloric intake from chow during intralipid presentations, we also measured 48-h chow intake during the final two 48-h tests. A pre-weighed amount of chow was presented before testing, and total intake, accounting for spillage, was measured at the completion of each 48-h test.

### Lingual Epithelium and Plasma Collection

Approximately 3 weeks after completion of two-bottle preference tests for oil emulsions, GF and NORM mice (n = 10 each) were sacrificed for collection of lingual epithelium and plasma after either a fast or re-feeding with intralipid. After an overnight-food deprivation (1700–0900-h), half of GF and NORM mice (n = 5 each) received a burette filled with 1-ml of 20% intralipid, while the other received a burette filled with water. Mice were sacrificed via decapitation 30-min after drinking the total volume of intralipid. Trunk blood was collected in EDTA-coated tubes (Becton Dickinson) containing 35 µl aprotonin (Sigma), 20 µl pefabloc (Sigma), and 20 µl DPP-4 inhibitor (Millipore), centrifuged at 3,500×*g* at 4°C, plasma aliquoted, and stored at −80° for further analysis. The posterior lingual epithelium was collected from fasted and re-fed mice by excising the tongue, and subdermally injecting 0.5 ml of 1 mg/ml dispase and elastase dissolved in mammalian physiological saline containing 1,2-Bis(2-Aminophenoxy)ethane-N,N,N′,N′-tetraacetic acid (Sigma, France). After 20-min incubation at room temperature, the posterior lingual epithelium containing the circumvallate papillae was dissected under a Stereoscope (Zeiss) and placed into a 1.5-ml microfuge tube containing AllProtect Tissue Reagent (Qiagen, France) and stored at 2°C.

### Intestinal Epithelial Cell Collection

For quantification of intestinal epithelial proteins, a separate group of GF and NORM mice (n = 5 per group) were used. Under deep isofluorane anesthesia, the proximal portion of the small intestine, containing the duodenum and jejunum was removed and placed into sterile physiological saline. Intestinal epithelial cells were collected using the everted sac method. Briefly, after excision, proximal intestines were flushed using 10 ml of ambient physiological saline followed by 10 ml oxygenated (95∶5 O_2_:CO_2_) Ca^+2^ and Mg^+2^-free Krebs-Heinslet buffer. After rinsing, intestines were everted, divided into three segments, and placed into flasks with oxygenated Ca+2, Mg+2-free Krebs-Heinslet buffer with EDTA and DTT. Flasks were placed in a 37°C water bath and shaken for 20 min to dissociate epithelial cells from the connective tissue. The subsequent suspension was collected, centrifuged and washed with sterile Dulbecco’s Phosphate Buffered-Saline without Ca^+2^ or Mg^+2^. This process was repeated three times. Aliquots of isolated intestinal cells were snap frozen and stored at −80°C.

### Quantitative Real-Time PCR (qRT-PCR)

Posterior lingual epithelium was lysed and homogenized using a TissueLyser (Qiagen, France) and RNA extracted using a RNEasy Fibrous Tissue Mini-kit (Qiagen, France) according to the manufacturer’s instructions. For cDNA synthesis, 2 µg of RNA was reverse transcribed in a reaction volume of 60 µl, using a high-capacity cDNA kit (Applied Biosystems, Courtaboeuf, France). Subsequent cDNA was diluted 5-fold and qPCR performed in a reaction volume of 20 µl using an ABI Prism 7700 (Applied Biosystems, Courtaboeuf, France) thermal cycler. Samples were run in triplicate and transcription levels of CD36 was quantified using Taqman® Gene Expression Assays and Gene Expression Master Mix (Applied Biosystems, Courtaboeuf, France). Relative mRNA expression was quantified using the 2^−ΔΔCT^ method with β-actin as internal control.

### Western Blotting

Isolated intestinal epithelial cell aliquots were thawed on ice and suspended in 1-ml of radioimmunoprecipitation assay (RIPA) buffer containing protease inhibitors (Sigma, France). Cells were lysed and homogenized and the resulting homogenate was centrifuged for 20-min at 14,000×g at 4°C. The protein concentration in the supernatant was determined with NanoDrop system (GE Healthcare). Soluble protein (100 µg) was then run on SDS-PAGE gels containing 10–12% acrylamide, transferred to nitrocellulose membranes, and probed with anti-CD36, GPR40, GPR120, GPR41, FIAF, PYY, GLP-1, and CCK (Santa Cruz Biotechnology) antibodies. Immune complexes were detected by chemiluminescence (GE Healthcare). Quantification was performed by scanning densitometry using ImageJ (NIH, Bethesda, MD, USA) against β-actin (Santa Cruz Biotechnology) as internal control.

### Plasma Analysis

Plasma was analyzed for glucose, triglycerides (TG), total cholesterol and total high-density lipoprotein (HDL) using an AU 400 automated biochemical analyzer (Olympus). Additionally, circulating levels of leptin, PYY, and acyl-ghrelin were determined using Enzyme-linked Immunosorbent Assays (Millipore, France) according to manufacturer’s instructions.

### Immunohistochemistry (IHC) of Enteroendocrine Cells

A separate group of overnight food deprived 10-wk old GF and NORM mice (n = 4 per group), were sacrificed, and 3 cm sections of the duodenum, jejunum, ileum, and colon were quickly removed, opened, pinned mucosal side up in agarose coated petri-dishes, and fixed with 4% formaldehyde overnight. Intestinal segments were stored in 75% ethanol, embedded in paraffin, and 4-µm-thick microtome cut sections mounted on glass slides were processed using standard procedures. After deparaffinizing and rehydrating, slides were placed in 6% hydrogen peroxide for 30 minutes, then blocked with PBS/3% BSA/2% goat serum for one hour. Sections were incubated overnight at 4°C with rabbit polyclonal antibody raised against chromogranin A (1∶200, Abcam, ab15160), washed, incubated for 1-h at room temperature with biotinylated donkey anti rabbit antibody (1∶400, Santa Cruz), incubated with a hematoxylin solution for nuclear staining, and processed using DAB (Dako) for 10–20 seconds. Sections were then dehydrated and mounted with DPX (Sigma), and examined under 100× microscope (Nikon) for enteroendocrine cell counts. Counting was performed manually by two individuals blinded to the treatment by observing five, non-overlapping microscopic areas from similar locations of each intestinal segment between GF and NORM mice.

### Statistical Analyses

Differences in bodyweight gain between groups from the start to the end of the experiment were analyzed with student’s t-test. Preference for intralipid were determined by the following formula: (48-h intake of intralipid)/(48-h intake of total fluid)*100 and subjected to one-way (group) repeated measures (rm) ANOVA. Additionally, 48-h acceptance (raw intake solution) as well as total calories consumed from intralipid were subjected to two-way (group × concentration) rmANOVA. To determine taste sensitivity to intralipid (concentration at which the animal first prefers tastant over water), we performed paired student’s t-test for each concentration within each group. The resulting values from Western blotting and qPCR, were analyzed using student’s t-test. Levels of plasma biochemical markers and satiety peptides were analyzed by two-way (group × treatment) ANOVA with Bonferroni post-hoc tests, where appropriate. Enteroendocrine cell counts were calculated for each intestinal segment of GF or NORM group as the total of all five microscopic fields, and analyzed by student’s t-test. For all statistical tests, differences were considered significant at α<0.05.

## Results

### Body Weight

There were no significant differences in weight gain between GF (0.8±0.5 g) and NORM (0.9±0.3) mice during the duration of the experiment.

### 48-h Two-bottle Oil Preference and Acceptance

**Figure 1:**
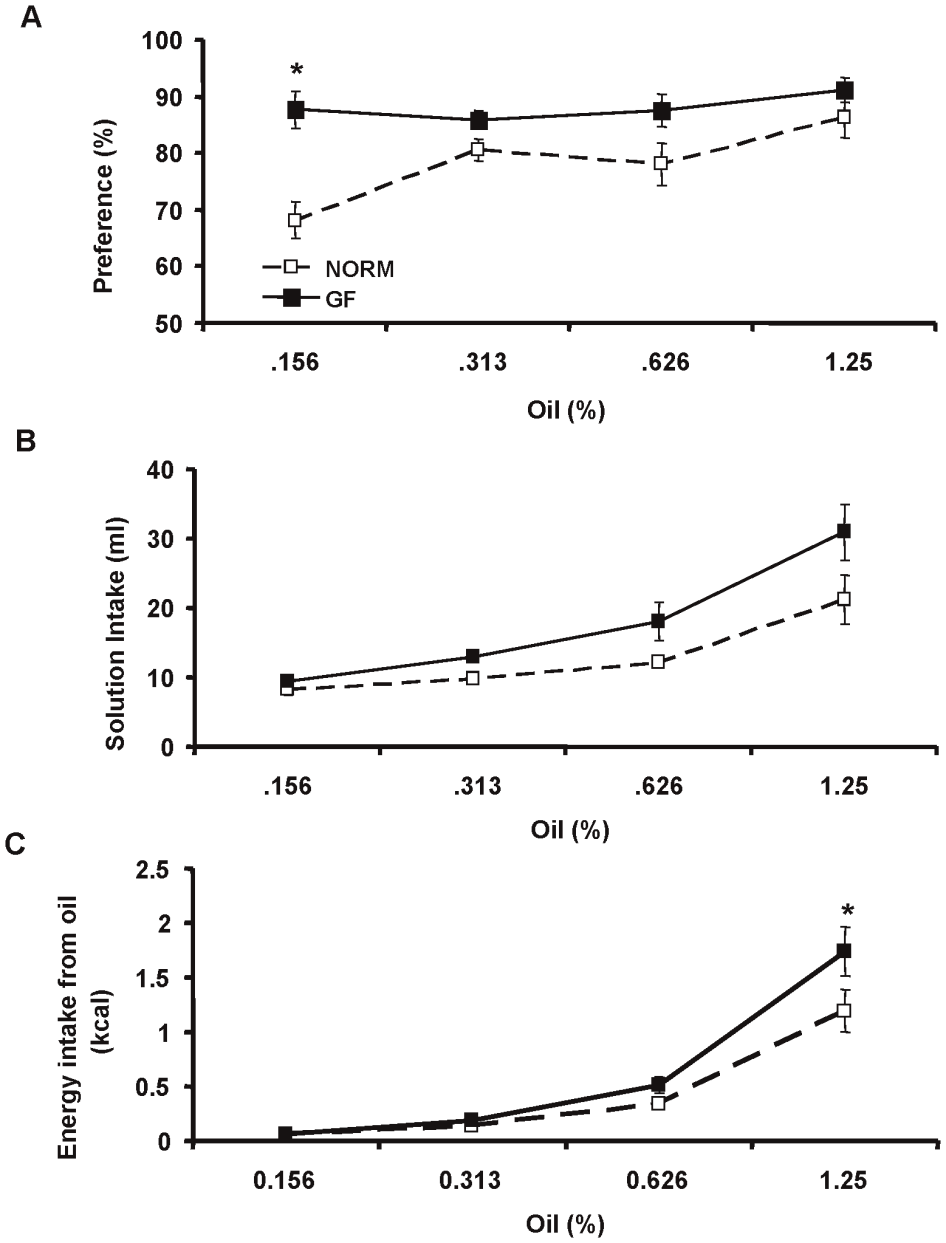
Preference (A), raw intake (B), and calorie intake from intralipid emulsions (C) in GF and NORM C57B6/J mice during 48-h two-bottle intralipid vs. water tests. (A) GF mice preferred the lowest concentration (0.156% oil) of intralipid emulsion test more than NORM mice. (B) Intake of intralipid emulsions was similar at each concentration tested, but increased overall in GF mice relative to NORM controls. (C) GF mice consumed more energy from the highest concentration (1.25% oil) of intralipid emulsion tested. Data are expressed as means±SEM. *P<0.05 compared to NORM.

There were significant main effects of concentration [F(3, 42) = 6.4, P = 0.01], group [F(1, 14) = 12.56, P<0.01], and group × concentration interaction [F(3, 42) = 3.8, P<0.05] on intralipid preference in GF and NORM mice. At the lowest concentration tested (0.156% oil), GF mice preferred intralipid to water more than NORM mice (GF: 87.72±3.2% vs. NORM: 68.09±3.3%; P<0.001) (Fig. 1A). When acceptance of 48-h intralipid intake was evaluated, there were significant main effects of concentration [F(3, 42) = 32.98, P<0.0001] and group [F(1, 14) = 5.66, P<0.05], but not group × concentration [F(3, 42) = 2.02, P = 0.13]. Thus, GF mice exhibited overall increased intralipid intake compared to NORM mice (Fig. 1B). However, when intake was converted into kilocalories, there was a significant main effect of concentration [F(3, 42) = 78.94, P<0.0001], and group × concentration interaction [F(3, 42) = 2.90, P<0.05], but not group [F(1, 14) = 4.43, P = 0.05]. Post-hoc analysis revealed a significant difference in caloric intake between GF (1.73±0.2 kcal) and NORM (1.19±0.2 kcal) mice at the highest concentration tested (1.25% oil) (P<0.05) (Fig. 1C). Additionally, we found no difference in 48-h solid chow energy intake between GF and NORM mice during exposure to 0.626% (GF: 10.14±0.3 kcal; NORM: 10.2±0.3 g) or 1.25% (GF: 10.04±0.5 g; NORM: 8.77±0.8 kcal) intralipid.

### Lingual and Intestinal CD36 Expression

**Figure 2:**
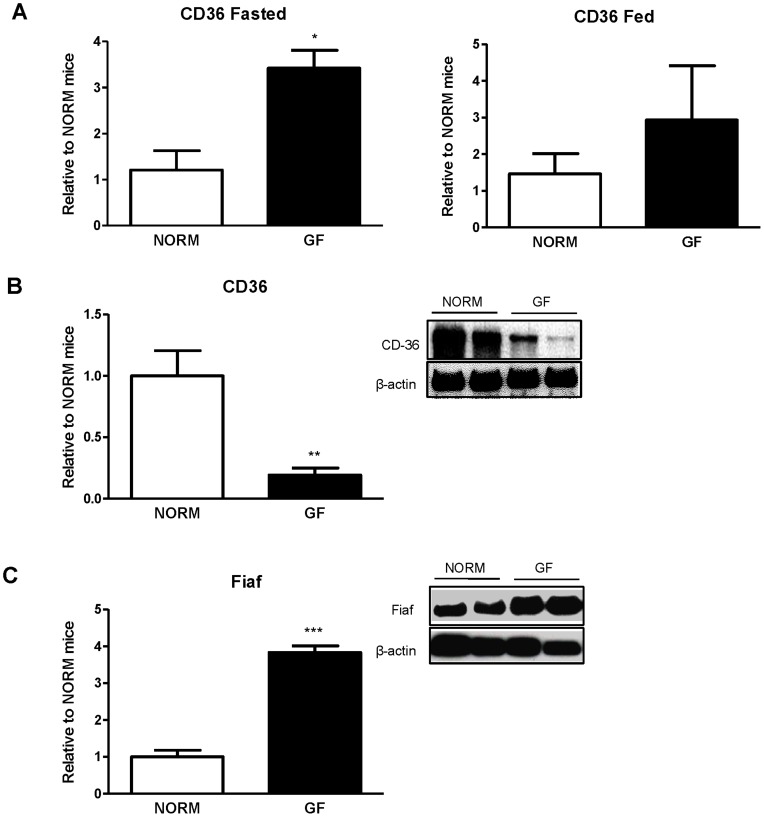
Gene expression of (A) lingual CD36, and protein expression of (B) intestinal CD36, and (C) intestinal FIAF. (A) GF mice exhibited 3-fold up-regulation of lingual CD36 mRNA in the posterior lingual epithelium relative to NORM mice during fasting. GF mice displayed a slight, but non-significant, increase in lingual CD36 expression following intralipid exposure. (B) Intestinal CD36 was significantly down-regulated in GF compared to NORM mice. (C) Intestinal FIAF expression was increased over 3-fold in GF relative to NORM mice Data are expressed as means±SEM. *P<0.05, **P<0.01, ***P<0.001, compared to NORM.

In GF mice, expression of CD36 transcript in the posterior lingual epithelium of fasted mice was up-regulated 3-fold relative to NORM mice (P<0.05) (Fig. 2A), with a similar, although non-significant, trend being observed after intralipid exposure (Fig. 2A). However, intestinal protein expression of CD36 was down-regulated in GF compared to NORM mice (P<0.05) (Fig. 2B). Additionally, intestinal FIAF expression in GF mice was up-regulated relative to NORM mice (P<0.001) (Fig. 2C).

### Intestinal Nutrient Receptor and Gut Peptide Protein Levels

**Figure 3:**
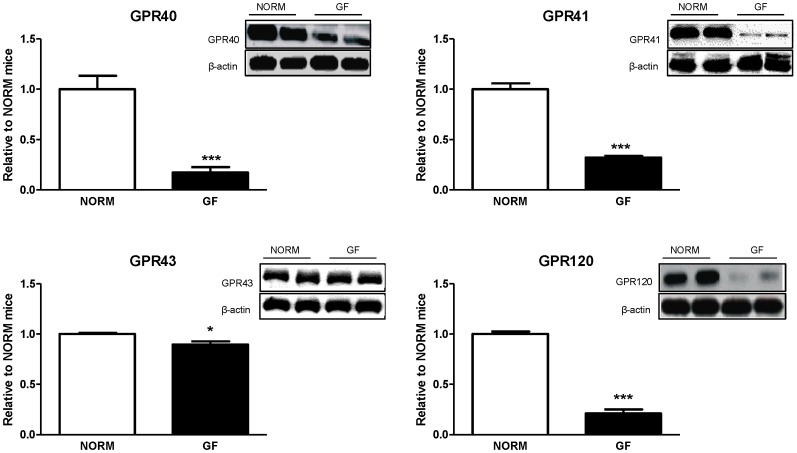
Intestinal epithelial protein expression of fatty-acid responsive receptors in GF and NORM mice. GF mice displayed down-regulation of proximal intestinal GPR40, 41, 43, and 120 relative to NORM mice. Data are expressed as means±SEM. *P<0.05, ***P<0.001, compared to NORM mice.

**Figure 4:**
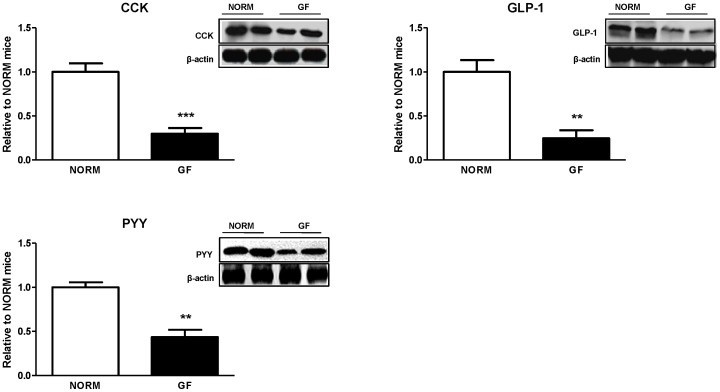
Intestinal epithelial protein expression of satiety peptide in GF and NORM mice. GF mice exhibited down-regulation of proximal intestinal CCK, GLP-1 and PYY relative to NORM mice. Data are expressed as means±SEM. **P<0.01, ***P<0.001, compared to NORM mice.

Protein expression of fatty-acid receptors GPR40 (P<0.0001), GPR41 (P<0.0001), GPR43 (P<0.05), and GPR120 (P<0.0001) in the proximal intestine was significantly decreased in GF mice relative to NORM controls (Fig. 3). Similarly, protein expression of CCK (P<0.0001), GLP-1 (P<0.001), and PYY (P<0.001) were also significantly decreased in GF compared to NORM mice (Fig. 4).

### Enteroendocrine Cell Counts

**Figure 5:**
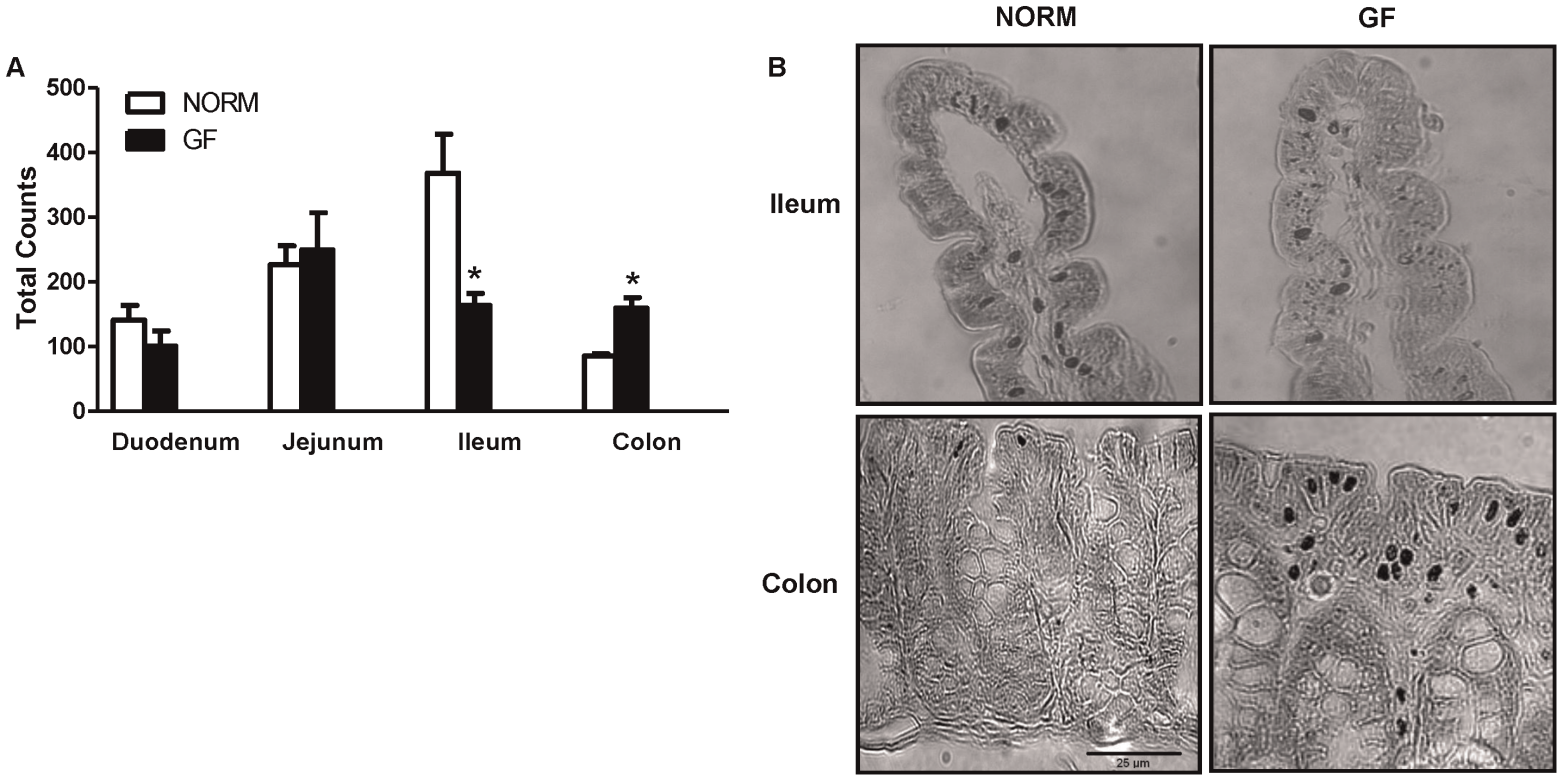
(A) Cell counts of enteroendocrine cells expressing chromogrannin-A and (B) representative microphotographs of ileum and colon sections at 100× magnification. GF mice had significantly less enteroendocrine cells in the ileum, but more in the colon, compared to NORM controls. Data are expressed as means±SEM. *P<0.05, compared to NORM mice.

Total enteroendocrine cells, represented by chromogranin-A stained cells, were increased in the colon (P<0.05), but decreased in the ileum (P<0.05) of GF compared to NORM mice (Fig. 5A-B). At the level of the duodenum and jejunum, there were no significant differences between groups.

### Plasma Analysis

**Figure 6:**
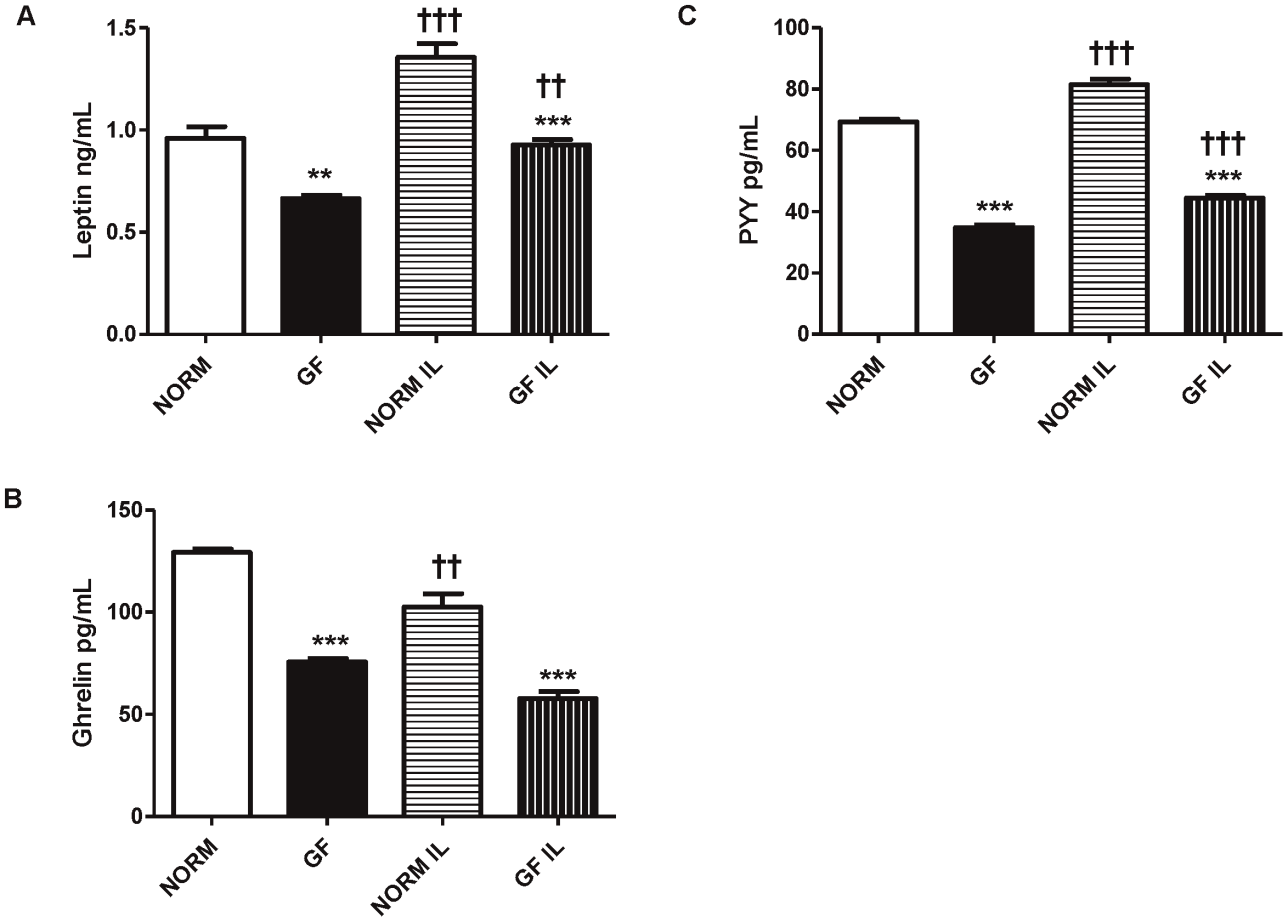
Plasma levels of (A) leptin, (B) ghrelin, and (C) PYY in GF and NORM mice following an overnight fast or re-feeding with 1-ml of 20% intralipid. (A) Plasma leptin was lower in GF mice relative to NORM controls. Re-feeding elevated plasma leptin in both groups. (B) GF mice displayed lower levels of plasma PYY compared to NORM mice while re-feeding increased plasma PYY in both groups. (C) GF mice exhibited lower circulating of ghrelin in both feeding conditions while re-feeding increased plasma ghrelin in NORM, but not GF mice. Data are expressed as means±SEM. **P<0.01 ***P<0.001, compared to NORM mice. ^††^P<0.01, ^†††^ P<0.001, compared to fasting condition within group.

Plasma gastrointestinal hormone levels were consistently decreased in GF mice compared to NORM controls. Specifically, GF mice had significantly lower levels of leptin in both fasted (P<0.001) and re-fed state (P<0.0001) compared to NORM mice, and re-feeding increased plasma leptin in both GF (P<0.001) and NORM (P<0.0001) mice (Fig. 6A). In both conditions, GF mice displayed decreased circulating PYY compared to NORM mice (P<0.0001 for both conditions) while re-feeding resulted in increased plasma PYY in both GF and NORM mice (P<0.0001 for both) (Fig. 6B). Ghrelin levels were also significantly lower in GF mice compared to NORM mice (P<0.0001 for both conditions); however, re-feeding decreased plasma ghrelin in NORM (P<0.001), but not GF mice (Fig. 6C).

**Figure 7:**
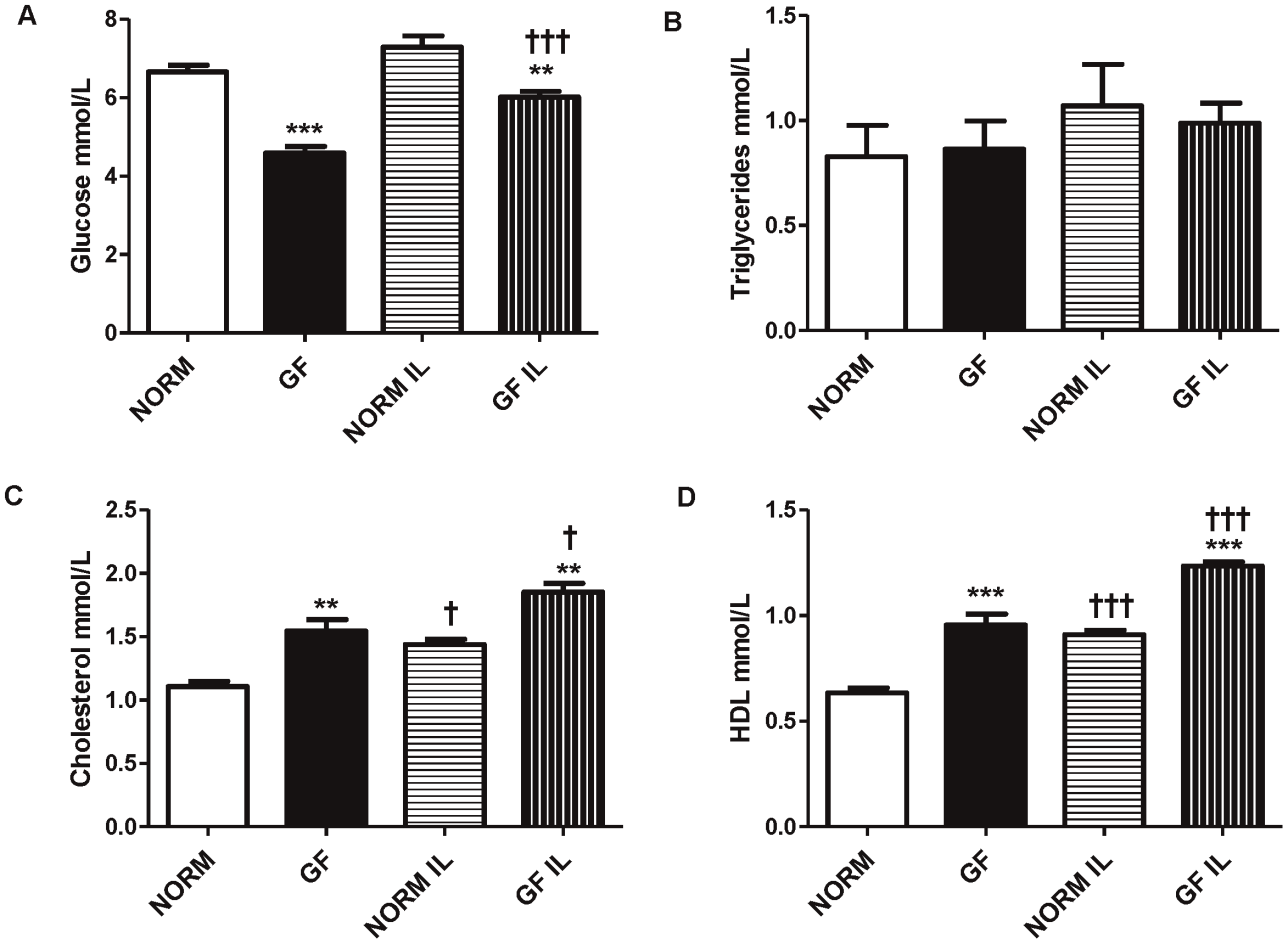
Plasma levels of (A) glucose, (B) total triglycerides, (C) cholesterol, and (D) HDL in GF and NORM mice following an overnight fast or re-feeding with 1-ml of 20% intralipid. (A) Plasma glucose levels of GF mice were lower than NORM controls and re-feeding increased glucose levels in GF, but not NORM mice. (B) Plasma triglycerides were similar between both groups and feeding conditions. (C) Plasma total cholesterol was increased in GF mice relative to NORM controls and re-feeding increased cholesterol levels in both NORM and GF mice. (D) Total plasma HDL was increased in GF mice relative to NORM mice with re-feeding increasing total HDL in both groups. Data are expressed as means±SEM. **P<0.01 mice, ***P<0.001 compared to NORM. ^†^P<0.05, ^††^ P<0.01, compared to fasting condition within group.

During both fasted (P<0.0001) and re-fed (P<0.001) conditions, GF mice had significantly lowers levels of glucose compared to NORM mice, however, re-feeding increased glucose levels in GF (P<0.0001), but not NORM mice (Fig. 7A). Triglyceride levels were similar between GF and NORM mice in both conditions (Figure 7B). Total cholesterol was increased in GF compared to NORM mice in both fasted (P<0.001) and re-fed (P<0.001) conditions. Additionally, total cholesterol levels were elevated after re-feeding in both GF (P<0.05) and NORM (P<0.05) mice compared to fasting (Fig. 7C). Consistent with this, HDL levels were significantly higher in GF mice in both conditions (fasted: P<0.0001; re-fed: P<0.0001) compared to NORM mice. Additionally, plasma HDL was elevated after re-feeding in both groups of mice (P<0.0001 for both) (Fig. 7D).

## Discussion

Our present studies demonstrate that GF mice display an increased preference for a low concentration of intralipid and consume slightly more intralipid than NORM mice, resulting in increased caloric intake. This increased preference for, and intake of, intralipid in GF mice is associated with increased expression of lingual CD36 and down-regulation of intestinal fatty-acid receptors. Furthermore, GF mice have decreased expression of intestinal satiety peptides CCK, GLP-1, and PYY and lower levels of circulating leptin, PYY and ghrelin. GF mice also have fewer enteroendocrine cells in the ileum, and more in the colon, but an equal number in the proximal (duodenum, jejunum) intestine, compared to NORM mice. Finally, GF mice display alterations in plasma biochemical markers that mimic a fasting state, with increased fat metabolism and decreased circulating glucose. Together, these results suggest that GF mice have increased oral but decreased post-oral nutrient detection and satiation signaling, contributing to increased energy intake, which most likely occurs as a compensatory mechanism for their decreased energy stores.

Oral and post-oral factors are strong determinants of meal size. For example, consumption of a HF-diet leading to increased fat metabolism in rodents is associated with increased acceptance of fat [5]. On the other hand, during the fasting state, lingual sensors for fat detection in the oral cavity are markedly increased [16]. Absence of the gut microbiota in mice results in a dramatic metabolic shift that closely resembles the fasting state of a normal animal [6]. For example, while body weight is similar between GF and normal mice, adiposity in GF mice is severely decreased, which is attributed to significant decreases in liver *de novo* lipogenesis [7]. Furthermore, plasma leptin and glucose are also lower in GF mice, an observation similar to that of a fasting state. These physiological and metabolic changes present in the GF condition may drive increased fat preference and/or intake observed in the current studies. Indeed, we found that GF mice prefer a low concentration of intralipid more than NORM mice while consuming more calories from intralipid at the highest concentration tested. These findings may be explained by the decreased energy status in the GF mice, leading to adaptive changes in the lingual epithelium, such as increased CD36. This is supported by the data showing that fasted animals exhibit increased preference for low concentrations of fats and increased caloric intake from fats [16]. Furthermore, our result of increased preference for the low intralipid concentration in GF mice was associated with increased expression of the fatty-acid translocase, CD36, in the posterior lingual epithelium during the fasted state. Expressed on the apical portion of sensory taste cells in the circumvallate papillae, CD36 plays a significant role in detection of long-chain fatty acids, and acts as a lipid sensor. For example, CD36 KO mice exhibit marked reduction in detection and preference for fats [15]. Additionally, expression of CD36 is elevated during fasting, a physiological state associated with increased oral sensitivity to fats [16], [18]. Conversely, HF-feeding and obesity is associated with decreases in lingual CD36 expression [16], although changes in lingual CD36 protein levels in obese mice has not been confirmed [19]. Thus, increased expression of CD36, leading to increased oral sensitivity to intralipid may be a secondary effect attributed to chronically depleted energy stores observed in GF mice, in agreement with previous data [6], [7]. After intralipid exposure, however, we found a modest and non-significant increase in lingual CD36 mRNA of GF mice relative to NORM controls. This suggests that, although the acute, limited caloric repletion had some influence on CD36 expression, similar to previous reports in fasted-refed conditions in normal mice [16], this small caloric load (2 kcal) was insufficient to compensate for the chronic energy depleted state of the GF status. As a result, CD36 mRNA expression of GF mice remained slightly elevated, compared to NORM mice even after intralipid exposure, still reflective of a fasting state. These results do not directly demonstrate a role for CD36 in the increased intralipid preference or consumption in GF animals, under normal feeding conditions. However, given that differences in intralipid preference were present at a relatively low concentration of intralipid, which provides minimal calories, it follows that oral, rather than intestinal factors influence its intake. This, together with the fact that CD36 might alter motivation for fat [19], could explain the increased preference for intralipid in GF mice. In addition to CD36, several other apically expressed proteins on the lingual epithelium also play a role in oral fat detection and possibly preference, which includes delayed rectifying potassium channels [20], [21], GPR40, and GPR120 [22]. Additionally, while it is unknown if microbiota in the oral cavity plays a role in taste signaling, increased expression of CD36 is most likely independent of changes in taste cell number. Specifically, we have previously found no difference in expression of α-gustducin, a marker of bitter and sweet taste receptor cells, T1R2, or T1R3 in the posterior lingual epithelium of GF and control mice [10]. However, despite the fact that GF mice are more sensitive to the low concentration of intralipid, intralipid is a nutritive fat source, and GF mice consume more calories from the high concentration of intralipid, denoting possible alterations in post-oral feedback.

While oral factors influence short-term preference and detection of stimuli, long-term acceptance and preference is predominantly driven by post-oral nutrient feedback, in addition to taste associations, which ultimately stimulate further consumption [23]. For example, intestinal infusions of nutrients paired with a flavored non-nutritive solution increases intake of that flavored solution [24]. As well, at higher concentrations of intralipid, CD36 KO mice display similar intralipid intake as wild type mice, and preference for nutritive fats is similar to wild type mice after repeated exposures with no impairments in post-oral conditioning. Thus, because GF mice have decreased energy stores and consume more of a nutritive solution than NORM counterparts, the composition and nutritive value of the intralipid, rather than oral factors, may be the main contributing factors for increased energy intake [15]. Although GF mice remain in a chronically fasting state, they also display a host of alterations in intestinal morphology and physiology. Specifically, GF animals have decreased intestinal villus length and crypth depth, lower rates of intestinal cell differentiation, all of which could contribute to impaired nutrient absorption [25]. Indeed, this is true of monosaccharide absorption, which is decreased in GF mice [6], and may be reflective of lower plasma glucose observed in our study. However, absorption of saturated fatty acids in GF mice is increased relative to controls [26], [27], which may be partially due to prolonged intestinal nutrient contact time as intestinal transit time is decreased in GF animals [11]. Finally, GF mice have decreased expression of intestinal CD36, which is predominantly located on the brush border; however, CD36 KO mice display no alterations in fat absorption [28]. Therefore, based on these data, it is unlikely that increased caloric intake in GF mice is due to decreased absorption of fats.

Enteroendocrine cells represent a candidate site of interaction between regulation of energy homeostasis and microbiota as they are exposed to the intestinal luminal environment, act as primary chemoreceptors, and respond to GI nutrients by releasing satiety peptides [29]. Emerging evidence has demonstrated that fatty-acid responsive GPRs located on enteroendocrine cells are responsible for secretion of gut peptides that control energy intake [30]. Furthermore, metabolic byproducts from the gut microbiota are thought to interact with some of these GPRs. Interestingly, GF animals display altered expression of intestinal nutrient receptors and associated changes in plasma intestinal satiety peptides [11], [31]. In the present study, we found decreased expression of fatty-acid receptors GPR40, 41, 43, and 120 in the proximal intestine of GF mice with parallel decreases in intestinal satiety peptide CCK, PYY, and GLP-1 expression that together may be responsible for increased energy intake in GF mice. While the majority of CCK is released from the proximal intestine, PYY and GLP-1 are predominately secreted from the L-cells located in the distal intestine. However, it has been shown that the duodenum contains enough L-cells capable of eliciting satiation through GLP-1 and PYY release [32]. Furthermore, unlike changes in the expression of CD36, which are most likely secondary adaptive responses, down-regulation of intestinal fatty-acid receptors seems to be a consequence of the lack of microbial stimulation. For example GPRs located on the luminal portion of enteroendocrine cells come into direct contact with the microbiota, which secrete nutritive byproducts of fermentation, and may alter nutrient receptor expression [11]. This is of relevance to our study, since secretion of GLP-1 and PYY from the proximal intestine is most likely a function of direct luminal nutrient stimulation, while distally released GLP-1 and PYY is primarily mediated by neural pathways [32]. It is known that consumption of fat or stimulation of intestinal cell lines with fatty acids results in release of satiety peptides such as CCK, PYY and GLP-1 through binding to GPR40, 41, 43, and 120. Specifically, short-chain fatty-acids (SCFA)-induced release of PYY is mediated by GPR41 and 43 [11], while GPR40 and 120 mediate CCK and GLP-1 secretion stimulated by medium-chain fatty acids and LCFAs, respectively [13], [30]. Very few studies have examined the relative influence of the gut microbiota on intestinal satiety peptides and nutrient receptors in the intestine, and no studies have linked these changes to appetitive responses. For example, Samuel et. al. found increased GPR41 in the colon of GF mice, which was associated with decreases in circulating PYY [11]. In our study, we only examined receptor expression in the proximal intestine and the relative distribution of fatty-acid responsive receptors throughout the GI tract is unclear. Our immunohistochemical data show no difference in the enteroendocrine number in the proximal intestine between GF and NORM mice. Thus, based on the broad decreases in the small intestinal GPRs and satiety peptide expression it appears that absence of microbiota affects intestinal peptide content rather than enteroendocrine cell numbers.

We also found that circulating levels of leptin, PYY, and ghrelin were all decreased in GF animals relative to controls. Although we have not assessed whole body fat composition in this study, carcasses of GF mice were virtually void of fat pads and we were unable to dissect any quantifiable fat depots from the GF mice. Because the majority of circulating leptin originates from white adipose tissue and GF mice are mostly fat depleted [6], decreased circulating leptin in GF mice is reflective of decreased adiposity. In addition, in a separate study we found that GF mice displayed drastically reduced fat mass (significantly less epididymal fat pad mass: GF: 0.04 g vs. NORM: 0.14 g; unpublished data) which was very similar to what we qualitatively observed in the mice from this current study. Our results are consistent with most previously published work demonstrating decreased body adiposity in GF C57Bl/6J mice. However, as mentioned in the Introduction, decreased adiposity was not observed in GF male adult C3H mice fed a HF-diet [9] which may be attributed to strain difference and the type of diet used. Thus with a 30% reduction in circulating leptin observed in our GF mice, a chronic energy deficit may be the main driving factor for increased caloric intake from intralipid. Similarly, PYY, which is released mainly from the distal intestine, where the majority of microbiota resides, is also decreased in GF mice. SCFA are potent stimulators of PYY release [33], thus it is not surprising that, decreased delivery of SCFA in the distal intestine, due to lack of the microbiota, results in decreased circulating PYY, similar to that proposed previously [11]. GF mice also had lower plasma levels of ghrelin compared to controls. As the only known orexigenic hormone released mainly from the stomach and duodenum, ghrelin is elevated during fasting and increases food intake and adiposity in rodent models when administered exogenously [34]. Based on this and given the constant energy deficits of the GF mice, one would expect increased circulating ghrelin in fasted GF mice. The reason for this effect is not immediately clear but changes in GI tract morphology, such as differences in X/A-cell number may be responsible. As expected, intralipid feeding increased leptin and PYY levels in both GF and NORM mice; however, re-feeding decreased ghrelin in NORM, but not GF mice, which may be reflective of the chronic fasting state in these animals.

In addition to changes in satiety hormone levels, we found slight alterations in circulating biochemical parameters. For example, plasma glucose was decreased in GF mice relative to NORM controls, an effect predictive of the energy deficits in the GF model and consistent with previous reports [7], [8]. Equally, we found that the intestinal glycoprotein FIAF, a lipoprotein lipase inhibitor, was significantly upregulated in GF mice. This is not unexpected, since intestinal microbiota promotes fat storage by suppressing intestinal expression of FIAF [6] and fasting increases FIAF expression [35]. However, the role of intestinal FIAF as an inhibitor of lipoprotein lipase in peripheral tissues of GF mice has been recently disputed [9]. FIAF stimulates lipolysis, resulting in elevated plasma triglycerides and lipoproteins with subsequent reduction in fat stores [36]. While we found no differences in total plasma TG levels, we found increases in plasma cholesterol and HDL in GF mice, consistent with the physical associations of FIAF with plasma lipoproteins [36]. Recent evidence suggests that serum TG levels are not altered in GF animals, but decreased LPL activity in this model has an effect on circulating TG levels [6], [37]. The reasons for the discrepancy in these findings regarding increased FIAF, yet unaltered plasma TG levels are not completely clear. While FIAF is indeed an important factor altering LPL activity in adipose tissue, recently, it has been suggested that intestinal FIAF levels do not influence circulating FIAF, as GF mice displayed increased intestinal FIAF but no difference in plasma FIAF compared to NORM mice [9]. Furthermore, FIAF is a potent inhibitor of angiogenesis [38], and gut microbiota has a profound ability to influence intestinal angiogenesis [39]. Thus, intestinal FIAF may serve as local contributor to angiogenesis rather than circulating metabolism. Additionally, cholesterol levels are typically unaltered or increased in GF rodents relative to controls during standard chow feeding [7], [37], [40], and decreased during HF-feeding [8]. Interestingly, increased circulating markers of fat metabolism are associated with increased acceptance of fat [5], supporting our behavioral findings. Together, these data confirms previous reports that markers of lipid metabolism are dramatically altered in GF animals and are influenced by energy status and feeding conditions.

In summary, we have shown that, under normal feeding conditions, GF mice prefer a low concentration of intralipid more than NORM mice, have increased overall intake and consume more calories from the high concentration of intralipid. This was associated with concomitant decreased expression of intestinal fatty-acid responsive receptors, decreased satiety peptide expression and decreased circulating levels of gut peptides. Furthermore, compared to NORM mice, GF mice had an increase in lingual CD36 mRNA expression after fasting, an effect that was diminished after feeding. As well, circulating biochemical markers indicated a shift toward increased fat metabolism in GF mice, while circulating satiety hormones signified decreased energy stores. Collectively, these results demonstrate, for the first time, that in addition to profound effects on energy status of the GF mouse resulting in a significant loss of adipose stores and subsequent metabolic changes, the absence of gut microbiota profoundly alters the physiological mechanisms and molecular substrates responsible for nutrient detection and signaling pathways that ultimately affect feeding behavior.
